# A nonparametric multiple imputation approach for missing categorical data

**DOI:** 10.1186/s12874-017-0360-2

**Published:** 2017-06-06

**Authors:** Muhan Zhou, Yulei He, Mandi Yu, Chiu-Hsieh Hsu

**Affiliations:** 1 Department of Epidemiology and Biostatistics, Mel and Enid Zuckerman College of Public Health, University of Arizona, 1295 N. Martin Ave., Tucson, 85724 USA; 2 Division of Research and Methodology, National Center for Health Statistics, Centers for Disease Control and Prevention, Hyattsville, 20782 USA; 3 Division of Cancer Control and Population Sciences, National Cancer Institute, Rockville, 20850 USA

**Keywords:** Categorical data, Double robustness, Missing at Random, Multiple imputation, Nearest neighbour

## Abstract

**Background:**

Incomplete categorical variables with more than two categories are common in public health data. However, most of the existing missing-data methods do not use the information from nonresponse (missingness) probabilities.

**Methods:**

We propose a nearest-neighbour multiple imputation approach to impute a missing at random categorical outcome and to estimate the proportion of each category. The donor set for imputation is formed by measuring distances between each missing value with other non-missing values. The distance function is calculated based on a predictive score, which is derived from two working models: one fits a multinomial logistic regression for predicting the missing categorical outcome (the outcome model) and the other fits a logistic regression for predicting missingness probabilities (the missingness model). A weighting scheme is used to accommodate contributions from two working models when generating the predictive score. A missing value is imputed by randomly selecting one of the non-missing values with the smallest distances. We conduct a simulation to evaluate the performance of the proposed method and compare it with several alternative methods. A real-data application is also presented.

**Results:**

The simulation study suggests that the proposed method performs well when missingness probabilities are not extreme under some misspecifications of the working models. However, the calibration estimator, which is also based on two working models, can be highly unstable when missingness probabilities for some observations are extremely high. In this scenario, the proposed method produces more stable and better estimates. In addition, proper weights need to be chosen to balance the contributions from the two working models and achieve optimal results for the proposed method.

**Conclusions:**

We conclude that the proposed multiple imputation method is a reasonable approach to dealing with missing categorical outcome data with more than two levels for assessing the distribution of the outcome. In terms of the choices for the working models, we suggest a multinomial logistic regression for predicting the missing outcome and a binary logistic regression for predicting the missingness probability.

**Electronic supplementary material:**

The online version of this article (doi:10.1186/s12874-017-0360-2) contains supplementary material, which is available to authorized users.

## Background

In population studies of public health, the health status of participants is a research outcome of interest and is commonly demonstrated using ordinal categories such as “Excellent”, “Good”, “Fair”, and “Poor”. However, these variables are typically subject to missing data. In practical analyses, researchers often create a specific new category for missing values (i.e., the missingness indicator approach) or use complete-case (CC) analysis, which excludes all missing observations. When missing values are missing at random (MAR), except for CC used to estimate regression coefficients, both methods are likely to induce substantial bias as well as lack of efficiency, e.g. to estimate the proportion of each category of the multinomial outcome variable, and therefore are not recommended in general [5, 9, 10].

The expectation-maximization (EM) algorithm uses the maximum likelihood approach to handle missing data problems. It consists of iterative expectation and maximization steps for estimating the parameter [4]. However, this approach only relies on the information from a working model predicting the missing values/outcome, and ignores the information embedded in missingness probabilities, that is, the probabilities of being missing (or nonresponse probabilities). Therefore the corresponding estimates might not be robust to certain misspecifications of the outcome model.

A more robust approach is to improve the estimation using additional information from the missingness probabilities, such as the use of calibration estimator (CE) [2], which is an extension of the inverse probability weighting method [7]. The estimator is a result of expressing the target parameter as a sum of two components, the model-based predictions and inverse probability-weighted prediction errors. These two components have a trade-off effect so that using their sum can achieve a doubly robust property. That is, the estimator remain consistent as long as one of the prediction models is correctly specified. In addition, CE uses covariates to fit working models for predicting missing values and missingness probabilities. Additional details about CE can be found in the “Methods” section.

Multiple imputation (MI) [13] is an attractive approach to missing data problems. It accounts for uncertainty in estimation due to missingness by imputing each missing observation multiple times [13, 15]. To the best of our knowledge, only a handful of studies have investigated the possible MI approaches to missing categorical data, such as MI using a loglinear model, MI using a latent class model, and MI using chained equations, [16, 20], and concluded that MI using a latent variable model had the best performance among the tested methods. However, we note that all these methods are only built on the working model predicting the missing values. An apparent disadvantage is that they might be sensitive to certain misspecifications of the working model.

To weaken the aforementioned, pure reliance on the working model for predicting the missing outcome, we propose a nearest neighbor-based MI (NNMI) approach to missing categorical data. The approach uses two working models, one for predicting missing outcome values (the outcome model) and the other for predicting the missingness probabilities using covariate information (the missingness model). Each working model is used to generate a predictive score. Their weighted sum is used to measure the “distance” between a missing case and observed cases. For each missing observation, its imputing set consists of observed cases which are “near” in terms of the distance function. The missing value can thus be imputed (replaced) by one of the donors in its imputing set. Because information from both the outcome and missingness models are used to impute the missing data, we surmise that the proposed method might possess some doubly-robust property.

Similar ideas have been proposed in [11] and [8]. In those contexts, the NNMI approach is applied to impute missing at random continuous variables and produces reasonable results under a variety of model misspecifications. However, a notable difference in our paper is that the NNMI approach is applied to MAR categorical variables. More specifically, if the number of categories is *M*, *M*−1 predictive scores can then be derived from the working model for the outcome, and a single predictive score can be derived from the working model for the missingness probabilities. It is of interest to investigate the optimal weighting schemes for the *M* predictive scores. We also note that there exist alternative ways of specifying models for categorical outcomes, which further complicates the problem. In addition, despite having a theoretical doubly robust property, CE might produce unstable results when missingness probabilities take some extreme values. It is of additional interest to assess the performance of NNMI in this scenario.

This article is organized as follows. In the “Methods” section, we specify notations, briefly introduce the CE method, and present the NNMI approach. In the “Simulation study” section, we investigate the properties of NNMI for finite samples. In the “Data example” section, we analyze a dataset from 2013 Behavioral Risk Factor Surveillance System (BRFSS) survey. Finally, we conclude our study with a discussion and suggest directions for future work.

## Methods

### Notation

Let *Y* denote the categorical outcome variable of interest with missing values, and suppose that *Y* has *M* categories of which the proportions need to be estimated. Let *δ* denote the missingness indicator, *δ*=0 if *Y* is missing and *δ*=1 if *Y* is observed. Let **X**=(*X*_1_,…,*X*_*p*_) denote a set of fully observed covariates that are predictive of *Y* and *δ*. Suppose that there are *n* independent subjects in the study.

### Calibration estimator

The calibration estimator is the earliest doubly robust method [2], which is based on two working models: one for the variable with missing values *Y*, and the other for the missingness indicator *δ*. For example, one can calculate the estimates of mean for a continuous *Y* by a sum of prediction and inverse probability-weighted prediction errors,

$$\mu=E[E(Y|\mathbf{X})]+E\left[\delta \frac{Y-E(Y|\mathbf{X}))}{\pi(\mathbf{X})}\right], $$

where *π*(**X**)=*E*(*δ*|**X**).

For a categorical *Y* taking values *m*=1,…,*M*, the estimator of probability that *Y*=*m* can be written as

$$\hat{P}_{CE,Y=m}\,=\,n^{-1}\sum\limits^{n}_{i=1} \hat{P}_{i,{Y=m}}+n^{-1}\sum\limits^{n}_{i=1}\delta_{i} w_{i} \left(I_{i,{Y=m}}\,-\,\hat{P}_{i,{Y=m}}\right)\!, $$

where $\hat {P}_{i,{Y=m}}$ is the predicted probability of *Y*=*m* based on, for example, a multinomial logistic regression model for *E*(*Y*|**X**) using complete cases, $w_{i}=1/\hat {\pi }(\textbf {X}_{i})$ is the inverse of the estimated probabilities of having case *i* being observed (computed using, for example, a logistic regression model from *E*(*δ*|**X**)), and *I* represents the indicator function of *Y*=*m*. The first term essentially predicts/imputes *Y* values using a model for *E*(*Y*|**X**) based on complete cases. The second term is a sum of prediction errors from the model for *E*(*Y*|**X**), adjusted by the inverse-probability weights using $\hat {\pi }(\mathbf {X})$ based on all cases. The doubly robust property dictates that the estimates for the probability of each category of *Y* would be consistent if at least one of the two models (*E*(*Y*|**X**) and *E*(*δ*|**X**)) is correctly specified.

### Nearest-neighbour multiple imputation

We first present the outcome model for *Y*. Given its categorical-feature, we consider two alternative modeling specifications as follows.

Specification I: In this generalized logit model (or multinomial logit model), without loss of generality, let *Y*=1 be the reference category. We model the ratio of each other category for *Y*=2,…,*M*, *Pr*(*Y*=*m*)/*Pr*(*Y*=1), (*m*=2,…,*M*), via the log link function and relate it to covariate **X**. That is,

$$\begin{array}{*{20}l} log\frac{Pr(Y=m)}{Pr(Y=1)}&=\boldsymbol{\alpha^{T}_{MLR,m}}\mathbf{X}_{O}, & (m=2, \ldots, M) \end{array} $$

where **X**_*O*_⊆**X** is a set of covariates predicting *Y*, and ***α***_***MLR,m***_=(*α*_*MLR,m*1_,…,*α*_*MLR,mp*_)^*T*^ is a vector of *p* regression coefficients for *Y*=*m* versus *Y*=1. Correspondingly, let

$$\begin{aligned} Z_{MLR,m}&=l_{m}\left(\mathbf{X}_{O}, \boldsymbol{\alpha_{MLR,1}}, \ldots, \boldsymbol{\alpha_{MLR,M-1}}\right) \\ &=\frac{exp\left(\boldsymbol{\alpha^{T}_{MLR,m}}\mathbf{X}_{O}\right)}{1\,+\,exp\! \left(\boldsymbol{\alpha^{T}_{MLR,1}}\mathbf{X}_{O}\right)\,+\,\ldots\,+\,exp\left(\boldsymbol{\alpha^{T}_{MLR,M-1}}\mathbf{X}_{O}\right)} \end{aligned} $$

be the predictive score of *Pr*(*Y*=*m*)/*Pr*(*Y*=1) under this generalized logit model. Therefore, there are *M*−1 predictive scores *Z*_*MLR,m*_ generated for *Y* with *M* categories, and the *m*-th score quantifies the “distance” between category *m* and category 1.

Specification II: An alternative approach is to fit *M*−1 cumulative logistic regression models. That is, models comparing *Y*∈(*m*+1,…,*M*) versus *Y*∈(1,…,*m*) for *m*=1,…,*M*−1. This model can be written as

$$log\frac{Pr(Y \in (m+1, \ldots, M))}{Pr(Y \in (1, \ldots, m))}=\boldsymbol{\alpha^{T}_{CLR,m}}\mathbf{X}_{O}, $$

where ***α***_***CLR,m***_=(*α*_*CLR,m*1_,…,*α*_*CLR,mp*_)^*T*^ is a vector of *p* regression coefficients for *Y*∈(*m*+1,…,*M*) versus *Y*∈(1,…,*m*). Under this model, each of the *M*−1 predictive scores is generated as

$$\begin{aligned} Z_{CLR,m}&=l_{m}\left(\mathbf{X}_{O},\boldsymbol{\alpha_{CLR,m}}\right)\\ &=\frac{exp\left(\boldsymbol{\alpha^{T}_{CLR,m}}\mathbf{X}_{O}\right)}{1+exp\left(\boldsymbol{\alpha^{T}_{CLR,m}}\mathbf{X}_{O}\right)}. \end{aligned} $$

Unlike specification I, there is no apparent constraint among *log*(*Pr*(*Y*∈(*m*+1,…,*M*))/*Pr*(*Y*∈(1,…,*m*))) for *m*=1,…,*M*−1. Therefore, the *m*-th score *Z*_*CLR,m*_ quantifies the “distance” between the consecutive categories *Y*∈(*m*+1,…,*M*) and *Y*∈(1,…,*m*).

Second, we present the model for missingness probabilities. It is common to use a logistic regression model for the missingness indicator *δ*. That is,

$$logit(Pr(\delta=1))=\mathbf{X}_{\delta}\boldsymbol{\beta}, $$

where **X**_*δ*_⊆**X** is a set of covariates predicting *δ* and ***β***=(*β*_1_,…,*β*_*p*_)^*T*^ is a vector of regression coefficients. The corresponding predictive score is

$$\begin{aligned} Z_{M} &=l_{M}\left(\mathbf{X}_{\delta},\boldsymbol{\beta}\right)\\ &=\frac{exp\left(\boldsymbol{\beta}^{T}\mathbf{X}_{\delta}\right)}{1+exp\left(\boldsymbol{\beta}^{T}\mathbf{X}_{\delta}\right)}. \end{aligned} $$

The aforementioned specifications do not encompass all possible modeling choices for *Y* and *δ*. For instance, the logit link functions can be replaced by probit or other link functions. In general, appropriate specifications should follow careful exploratory analysis and model diagnostics of the real data. Here we only use these specifications for illustrative purposes.

The general strategy of NNMI works as follows. The aforementioned predictive scores ({*Z*_*MLR,m*_} or $\{Z_{CLR, R_{m}}\}$ and *Z*_*M*_) can be calculated for each observation, missing *Y* or not. They are then standardized so that the effect from each covariate can be summarized on approximately the same scale. A weighted sum of the standardized scores is generated to balance the contribution from the two working models. This (weighted) predictive score is then used to quantify the “distance” between subjects. The scores from cases with observed *Y* are used to form imputing sets for subjects with missing *Y*. For each incomplete subject, imputations are randomly drawn from subjects (in the imputing set) that have smaller “distances” to the subject with a missing *Y*. To ensure the “properness” of multiple imputation [13], the estimation of scores and formation of imputing sets are conducted on bootstrap samples of the original data set to incorporate parameter uncertainty.

More specifically, the procedure consists of the following steps:

**Step 1: Bootstrap –** Bootstrap the original data set (including the missing observations) {**Y**,**X**} to obtain the bootstrap sample {**Y** ^∗^,**X** ^∗^}.

**Step 2: Calculating the predictive scores –** From the bootstrap sample we estimate the regression coefficients for the outcome and missingness models using the maximum likelihood method. For illustration suppose we use specification I for the outcome model and denote the corresponding regression coefficients as $\boldsymbol {\alpha ^{*}_{MLR,m}}, m=1, \ldots M-1$. Let ***β*** ^***∗***^ be the regression coefficients for the propensity model. We use these regression coefficients and the original covariate **X** to form the scores. For example, the *M*−1 predictive scores for the outcome model (under specification I) are

$$ \begin{aligned} Z_{MLR, m}^{*} & =l_{m}\left(\mathbf{X}_{O}, \boldsymbol{\alpha_{MLR,1}^{*}}, \ldots, \boldsymbol{\alpha_{MLR,M-1}^{*}}\right) \\ &=\frac{exp\left(\boldsymbol{\alpha^{*T}_{MLR,m}}\mathbf{X}_{O}\right)}{1\,+\,exp\!\left(\! \boldsymbol{\alpha^{*T}_{MLR,1}} \mathbf{X}_{O}\!\right)\,+\,\ldots\,+\,exp\left(\!\boldsymbol{\alpha^{*T}_{MLR,M-1}}\mathbf{X}_{O}\right)\! },\\ &\quad(m=1, \ldots, M-1). \end{aligned} $$

The predictive score for the propensity model is

$$\begin{aligned} Z_{M}^{*} &=l_{M}\left(\mathbf{X}_{\delta},\boldsymbol{\beta^{*}}\right)\\ &=exp\left(\boldsymbol{\beta^{*T}} \mathbf{X}_{\delta}\right)/\left(1+exp\left(\boldsymbol{\beta^{*T}}\mathbf{X}_{\delta}\right)\right). \end{aligned} $$

Each of the *M* predictive scores ${\{Z_{MLR, 1}^{*},\! \ldots \!, Z_{MLR, M-1}^{*},} Z_{M}^{*}\}$ is standardized by subtracting its mean and dividing by its standard deviation. The resulting standardized scores are denoted by **S**≡(*S*_1_,…,*S*_*M*_).

**Step 3: Forming the imputing set –** We calculate a distance function to define the similarity between subject *i* with missing *Y* in the original data set and subject *j* with observed *Y* in the bootstrap sample based on the *M* predictive scores, *S*_1_,…,*S*_*M*_. Specifically, the distance between subjects *i* and *j* is defined as

$$d(i,j)\,=\,\sqrt{\omega_{1}\! \left[S_{1}(i)-S_{1}(j)\right]^{2}+\ldots+\omega_{M} \left[S_{M}(i)-S_{M}(j)\right]^{2}}, $$

where *ω*_1_,…,*ω*_*M*_ are non-negative weights for the predictive scores, satisfying ${\sum \nolimits }^{M}_{i=1}\omega _{m}$=1. The way to calculate the similarity between subject *i* with missing *Y* in the original data and subject *j* with observed *Y* in the bootstrap sample was initiated from predictive mean matching and was described by Heitjan and Little [6], which was an extension of a method by Rubin [14]. Morris and his colleagues also studied on this nonparametric MI method compared with other methods [12]. The imputing set for subject *i* with a missing *Y* in the original data is the *NN* nearest neighborhood (i.e., the number of donors, a positive integer specified prior to imputation), *R*(*i,NN*,*ω*_1_,…;*ω*_*M*_), consisting of *NN* subjects with observed *Y* in the bootstrap sample and having the smallest *NN* distances *d*(*i,j*).

**Step 4: Imputation –** From the imputing set *R*(*i,NN*,*ω*_1_,…;*ω*_*M*_), an observation is randomly drawn (with equal probability) to replace the missing *Y* in subject *i*. This imputation is conducted for all *i*’s. Once all missing observations of *Y* are imputed, one fully imputed data set is obtained. Return to Step 1.

**Step 5: Analyzing multiply imputed data sets –** Steps 1 to 4 are independently repeated *K* time to obtain *K* imputed data sets for estimation. For each imputed data set, an estimate of the probability of *Y*=*m* is calculated by $\hat {P}_{\hat {Y}=m}=n_{m}/n, m=(1, \ldots, M)$, where *n*_*m*_ is the number of $\hat {Y}=m$ observed in the imputed data set and *n* is the sample size. Denote $\hat {P}_{\hat {Y}=m}(k)$ as the estimate for the *k*-th imputed data set. Using Rubin’s combining rules [10], the final NNMI estimator is the average across *K* imputed data sets as $\bar {P}_{{\hat {Y}=m}}=(1/K)\sum _{k=1}^{K} \hat {P}_{\hat {Y}=m}(k)$. Its variance can be estimated using the sum of a between-imputation and within-imputation component as

$${{\begin{aligned} SE\!\left(\bar{P}_{\hat{Y}=m}\right)\,=\,\!\sqrt{\!\frac{1}{K}\!\sum\limits_{k}\! s^{2}_{\hat{Y}=m}(k) \,+\, \left(1\,+\,\frac{1}{K}\right)\!\!\left(\frac{1}{K\,-\,1}\right)\!\!\sum\limits_{k}\!\left(P_{\hat{Y}=m}(k) \,-\,\bar{P}_{\hat{Y}=m}\right)^{2}}, \end{aligned}}} $$

where $s_{\hat {Y}=m}(k)$ is the standard error for the probability of $\hat {Y}=m$ in the *k*-th data set based on a Bernoulli distribution for the event $I(\hat {Y}=m)$.

We use R to perform all the simulations and data analysis. Multinomial logistic regression models can be done using the multinom function from the nnet package of R. [17].

## Results

### Simulation study

Past literature [8, 11] has suggested that the use of two working models for predicting missing values and missingness probabilities in NNMI can induce a double robustness property when *Y* is continuous. We surmise that would hold as well when *Y* is categorical. This section presents a simulation study for assessing the performance of the NNMI under some model misspecifications for imputing a three-category nomial missing outcome and evaluating the estimation of proportions of all three categories.

For simplicity, we consider an incomplete trichotomous outcome *Y* (i.e. *M*=3). The estimand of evaluation is the probability *P*(*Y*=*m*), *m*=1,2,3. The methods compared include: fully observed (FO) analysis, which is treated as the gold standard because the analysis is applied before some of the *Y*s are removed; complete-case (CC) analysis, which excludes cases with missing *Y*; the calibration estimator (CE); a parametric MI (PMI), which imputes the missing values by taking the predictive values from a multinomial logistic regression model for the missing values; and the proposed NNMI approach. For the latter approach, the method using multinomial logistic regressions for the outcome model is denoted as *NNMI*_*MLR*_(*NN*,*ω*_1_,…;*ω*_*M*_), and that using cumulative logistic regressions is denoted as *NNMI*_*CLR*_(*NN*,*ω*_1_,…;*ω*_*M*_). The previous work on NNMI [8, 11] has demonstrated that bias increases while SD and SE decreases when NN increased. It is suggested that NN= 3 or 5 in general result in slightly lower MSEs. In the simulation, *NN*=5 is chosen for NNMI. Based on our previous experience, ten-time MI is usually sufficient to control for the uncertainty. In this article, we compared one table using K=10 and K=50 (Table 1 and Additional file 1: Table S12). The results shows no clear difference in bias and slightly lower SD and SE using K=50.

**Table 1:** Simulation results from probability estimation for *Y*, where *Y* is generated using a logit link function with five covariates, *δ* is generated using a logit link function with not extreme missingness probabilities (M1) based on five covariates, N = 400

	*Pr*(*Y*=1)=0.386				*Pr*(*Y*=2)=0.288			
Method	Est	SD	SE	CR	Est	SD	SE	CR
FO	0.386	0.023	0.024	0.960	0.286	0.023	0.023	0.934
CC	0.439	0.034	0.035	0.674	0.340	0.034	0.033	0.670
	Working models for *Y*:				Five covariates with logit link			
	Working models for *δ*:				Five covariates with logit link			
CE	0.388	0.036	0.036	0.948	0.286	0.038	0.036	0.924
PMI	0.387	0.030	0.032	0.954	0.287	0.034	0.032	0.930
*NNMI*_*MLR*_(5,0.4,0.4;0.2)	0.387	0.032	0.033	0.952	0.288	0.036	0.033	0.936
*NNMI*_*MLR*_(5,0.1,0.7;0.2)	0.389	0.033	0.034	0.956	0.288	0.035	0.033	0.930
*NNMI*_*MLR*_(5,0.7,0.1;0.2)	0.386	0.032	0.033	0.956	0.290	0.036	0.034	0.926
*NNMI*_*CLR*_(5,0.4,0.4;0.2)	0.385	0.032	0.033	0.948	0.294	0.036	0.034	0.916
*NNMI*_*CLR*_(5,0.1,0.7;0.2)	0.381	0.032	0.033	0.944	0.295	0.037	0.034	0.928
*NNMI*_*CLR*_(5,0.7,0.1;0.2)	0.390	0.032	0.033	0.950	0.294	0.037	0.034	0.936
	Working models for *Y*:				Three covariates with logit link			
					(misspecified scenario 1)			
	Working models for *δ*:				Five covariates with logit link			
CE	0.311	0.057	0.057	0.760	0.288	0.041	0.041	0.932
PMI	0.464	0.037	0.038	0.454	0.285	0.032	0.031	0.922
*NNMI*_*MLR*_(5,0.4,0.4;0.2)	0.410	0.036	0.039	0.932	0.290	0.035	0.033	0.926
*NNMI*_*MLR*_(5,0.1,0.7;0.2)	0.407	0.036	0.039	0.940	0.290	0.035	0.033	0.932
*NNMI*_*MLR*_(5,0.7,0.1;0.2)	0.408	0.035	0.039	0.930	0.291	0.035	0.033	0.928
*NNMI*_*CLR*_(5,0.4,0.4;0.2)	0.415	0.036	0.038	0.896	0.292	0.034	0.033	0.940
*NNMI*_*CLR*_(5,0.1,0.7;0.2)	0.412	0.036	0.039	0.916	0.292	0.035	0.033	0.934
*NNMI*_*CLR*_(5,0.7,0.1;0.2)	0.413	0.035	0.039	0.926	0.291	0.035	0.034	0.954
	Working models for *Y*:				Five covariates with logit link			
	Working models for *δ*:				Three covariates with logit link			
					(misspecified scenario 2)			
CE	0.389	0.032	0.033	0.954	0.285	0.033	0.032	0.942
PMI	0.387	0.030	0.032	0.954	0.287	0.034	0.032	0.930
*NNMI*_*MLR*_(5,0.4,0.4;0.2)	0.393	0.032	0.033	0.962	0.292	0.035	0.033	0.936
*NNMI*_*MLR*_(5,0.1,0.7;0.2)	0.402	0.034	0.035	0.936	0.289	0.035	0.033	0.926
*NNMI*_*MLR*_(5,0.7,0.1;0.2)	0.389	0.031	0.033	0.960	0.297	0.036	0.034	0.936
*NNMI*_*CLR*_(5,0.4,0.4;0.2)	0.387	0.031	0.032	0.958	0.298	0.035	0.033	0.936
*NNMI*_*CLR*_(5,0.1,0.7;0.2)	0.382	0.031	0.033	0.956	0.298	0.035	0.034	0.940
*NNMI*_*CLR*_(5,0.7,0.1;0.2)	0.392	0.031	0.033	0.954	0.302	0.035	0.034	0.920

Est: Estimates of probabilities; SD: Empirical standard deviation; SE: Estimate of standard error; CR: Coverage rate of 95% confidence intervals; FO: fully observed; CC: Complete Cases; CE: Calibration estimator; PMI: Parametric Multiple Imputation; *NNMI*_*MLR*_(*NN*,*ω*_1_,*ω*_2_;*ω*_3_): the NNMI method using Multinomial Logistic Regressions, NN is the number of nearest neighbors and weights are *ω*_1_,*ω*_2_, and *ω*_3_; *NNMI*_*CLR*_: the NNMI method using Cumulative Logistic Regressions; *K* = 10 imputed datasets are used for PMI and NNMI methods

Sample size *n*=400 and *n*=200 are considered for simulation. For each tested scenario, the simulation is conducted 500 times. The criteria include the average estimate (EST), the empirical standard deviation (SD), the average standard error (SE), and the coverage rate (CR) of 95% confidence intervals (CI), all of which are calculated from the 500 simulations. To assess the performance of the simulations, Monte Carlo Errors (MCEs) are calculated for each measure using the formulas from White’s paper [19]. In FO and CC, the SEs are calculated assuming a Bernoulli distribution for $\hat {Y}=m$. In CE, the SE’s are calculated using bootstrap. In PMI and NNMI, Rubin’s combining rules are used to calculate SE’s.

Five observed covariates **X**=(*X*_1_,…,*X*_5_) are independently generated from *U*(−1,1). The trichotomous outcome, *Y*, is generated from a multinomial distribution with probabilities as *Pr*(*Y*=1|**X**)=*g* ^−1^(*X*_1_−*X*_2_+2*X*_3_−2*X*_4_+5*X*_5_),*Pr*(*Y*=2|**X**)=*g* ^−1^(2*X*_1_−2*X*_2_+3*X*_3_−3*X*_4_+1.5*X*_5_) and *Pr*(*Y*=3|**X**)=1−*Pr*(*Y*=1|**X**)−*Pr*(*Y*=2|**X**), where *g* is a link function. We consider both the logit and probit link functions for *g*.

We assume that *Y* is independent of *δ* given **X**. That is, the missingness probability of *Y* is only dependent on **X** (i.e., MAR). Two models are considered for the missingness indicator: *Pr*(*δ*=1|**X**)=*g* ^−1^(0.5*X*_1_−*X*_2_+*X*_3_−*X*_4_+*X*_5_) (denoted M1); and *Pr*(*δ*=1|**X**)=*g* ^−1^(0.5*X*_1_+2*X*_2_−4*X*_3_−2*X*_4_+2*X*_5_) (denoted M2). Again, we consider both the logit and probit link functions for *g*. In general, Model M1 generates missingness probabilities that are mostly bounded away from 1 or 0 with a bell-shape distribution, whereas Model M2 generates more missingness probabilities that are close to 1 or 0 with a U-shape distribution. That is, the missingness probabilities generated from model M2 are more extreme than those from model M1. In both schemes, the overall missingness probability is approximately 50%.

The primary goal of the simulation is to assess the performance of the NNMI various misspecifications of the working models for *Y* and *δ*. In general, two types of model misspecification for working models are tested: 1) including a reduced set of predictors (i.e., *X*_1_,*X*_2_, and *X*_3_) for working models; and 2) misspecified link functions for working models. More specifically, we consider the following five scenarios:

- Scenario 1 Misspecified working model for *Y* only: including only 3 predictors and using a correct link function
- Scenario 2 Misspecified working model for *δ* only: including only 3 predictors and using a correct link function
- Scenario 3 Misspecified working model for *Y* only: using an incorrect link function and including all 5 predictors
- Scenario 4 Misspecified working model for *δ* only: using an incorrect link function and including all 5 predictors
- Scenario 5 Misspecified working models for both *Y* and *δ*: using incorrect link functions for both and including all 5 predictors

For example, we firstly simulate *Y* using *X*_1_−*X*_5_ through logit link and simulate *δ* using *X*_1_−*X*_5_ through logit link. The misspecification Scenario 1 fit a working model for *Y* with same logit link function but using *X*_1_,*X*_2_ and *X*_3_ only, and fit a working model for *δ* using same five covariates, *X*_1_−*X*_5_, and same logit link function.

We also assess the effects of the extremeness of missingness probabilities on the performance of the methods. This is motivated by the fact that CE, despite being theoretically consistent if one of the working models is correctly specified, might have unstable results when the propensity model generates extreme missingness probabilities.

Another aim of the simulation is to investigate the optimal strategy for specifying weights *ω*_*m*_’s. Previous literature [8, 11] has suggested that a small, non-zero weight is often necessary for the score from the missingness probability model if *Y* is continuous. Because *M*=3 here in our setting, we consider several combinations for (*ω*_1_,*ω*_2_;*ω*_3_) because these weights might reflect the applicant’s belief on the validity of the three working models. Our investigation results (not shown) indicate that NNMI performs well when the weight for the score from the missingness probability model (*ω*_3_) is non-zero, consistent with conclusions from [8, 11]. In addition, positive weight(s) for the scores from the working model(s) for predicting missing values (*ω*_1_ and *ω*_2_) are necessary when only the working model for the missingness probability is misspecified. Therefore, the following simulation results present the results from fixing *ω*_3_=0.2 and different combinations for *ω*_1_ and *ω*_2_: (0.4,0.4;0.2), (0.7,0.1;0.2), and (0.1,0.7;0.2).

For brevity, we only present and illustrate the results from *n*=400. With smaller sample size of 200 observations (See Additional file 1), SEs from all methods slightly increase as expected, and the major comparative pattern does not vary dramatically. Most of the MCEs are less than 5%.

Table 1 summarizes the results when logit link functions are used to generate *Y* and *δ*. In addition, the missingness probabilities are generated by Model M1 and do not have many extreme values. As expected, CC estimates have substantial biases and the coverage rates are low. When the working models for *Y* and *δ* are both correctly specified, the bias is negligible for CE. PMI yields a good performance as well. *NNMI*_*MLR*_ produces slightly larger biases yet smaller SD compared with CE. *NNMI*_*CLR*_ produce slightly worse yet comparable results. When only the working model for *Y* is misspecified with 3 covariates, both NNMI methods are superior to other methods in term of bias and coverage rate, apparently for estimating *Pr*(*Y*=1). In this case, CE estimates have much larger biases and variations, as well as low CRs. PMI breaks down dramatically due to its sole reliance on the working model predicting *Y*. The methods are more or less similar for estimating *Pr*(*Y*=2). When only the working model for *δ* is misspecified with 3 covariates, CE performs well. Both NNMI produce mostly comparable results with CE.

Table 2 presents the results when logit link functions are used to generate *Y* and *δ*. Here more extreme missingness probabilities are generated by Model M2. Compared with Table 1, the performances of estimators degrade in general due to the fact that these extreme missingness probabilities are more difficult to estimate and thus render more instability to the estimates. When both working models are correctly specified, CE produces little bias yet extremely large SD and SE. Both NNMI methods have small biases which lead to lower-than-nominal CRs. However, their variations (SD) and the estimates (SE) are much smaller and more reasonable compared with CE. Between the two approaches, *NNMI*_*CLR*_ is somewhat inferior to *NNMI*_*MLR*_. When only the working model for *Y* is misspecified with 3 covariates, CE estimates become largely biased for estimating *Pr*(*Y*=1). In this case, however, both NNMI methods produce smaller biases and much lower SDs and SEs. When only the working model for *δ* is misspecified with 3 covariates, interestingly CE works better than NNMI methods. We surmise this is due to the fact that, although misspecified, the working propensity model for CE avoids most of the extreme missingness probabilities. In other words, a correctly specified working propensity model might do more harm (i.e., brings more variation to the estimates) to CE compared with NNMI if the missingness probabilities are more extreme.

**Table 2:** Simulation results from probability estimation for *Y*, where *Y* is generated using a logit link function with five covariates, *δ* is generated using a logit link function with extreme missingness probabilities (M2) based on five covariates, N = 400

	*Pr*(*Y*=1)=0.386				*Pr*(*Y*=2)=0.288			
Method	Est	SD	SE	CR	Est	SD	SE	CR
FO	0.386	0.023	0.024	0.960	0.286	0.023	0.023	0.934
CC	0.425	0.031	0.033	0.802	0.374	0.033	0.033	0.250
	Working models for *Y*:				Five covariates with logit link			
	Working models for *δ*:				Five covariates with logit link			
CE	0.378	0.102	0.080	0.946	0.288	0.108	0.076	0.902
PMI	0.385	0.034	0.036	0.950	0.288	0.036	0.033	0.922
*NNMI*_*MLR*_(5,0.4,0.4;0.2)	0.389	0.039	0.040	0.946	0.297	0.043	0.040	0.906
*NNMI*_*MLR*_(5,0.1,0.7;0.2)	0.399	0.042	0.045	0.942	0.292	0.041	0.039	0.918
*NNMI*_*MLR*_(5,0.7,0.1;0.2)	0.385	0.038	0.039	0.936	0.302	0.044	0.042	0.916
*NNMI*_*CLR*_(5,0.4,0.4;0.2)	0.384	0.037	0.038	0.938	0.304	0.042	0.040	0.918
*NNMI*_*CLR*_(5,0.1,0.7;0.2)	0.372	0.038	0.039	0.926	0.307	0.043	0.042	0.918
*NNMI*_*CLR*_(5,0.7,0.1;0.2)	0.395	0.038	0.039	0.944	0.305	0.043	0.041	0.908
	Working models for *Y*:				Three covariates with logit link			
					(misspecified scenario 1)			
	Working models for *δ*:				Five covariates with logit link			
CE	0.302	0.234	0.184	0.946	0.287	0.117	0.084	0.910
PMI	0.495	0.039	0.042	0.258	0.288	0.032	0.031	0.932
*NNMI*_*MLR*_(5,0.4,0.4;0.2)	0.436	0.051	0.053	0.852	0.295	0.042	0.040	0.914
*NNMI*_*MLR*_(5,0.1,0.7;0.2)	0.431	0.052	0.054	0.878	0.293	0.041	0.040	0.932
*NNMI*_*MLR*_(5,0.7,0.1;0.2)	0.433	0.050	0.053	0.858	0.296	0.042	0.041	0.924
*NNMI*_*CLR*_(5,0.4,0.4;0.2)	0.440	0.047	0.049	0.806	0.297	0.040	0.039	0.924
*NNMI*_*CLR*_(5,0.1,0.7;0.2)	0.429	0.046	0.048	0.852	0.299	0.042	0.039	0.926
*NNMI*_*CLR*_(5,0.7,0.1;0.2)	0.441	0.050	0.051	0.806	0.297	0.041	0.040	0.920
	Working models for *Y*:				Five covariates with logit link			
	Working models for *δ*:				Three covariates with logit link			
					(misspecified scenario 2)			
CE	0.386	0.050	0.048	0.960	0.286	0.043	0.040	0.894
PMI	0.385	0.034	0.036	0.950	0.288	0.036	0.033	0.922
*NNMI*_*MLR*_(5,0.4,0.4;0.2)	0.398	0.038	0.040	0.952	0.301	0.041	0.039	0.906
*NNMI*_*MLR*_(5,0.1,0.7;0.2)	0.426	0.042	0.045	0.858	0.294	0.039	0.037	0.922
*NNMI*_*MLR*_(5,0.7,0.1;0.2)	0.392	0.037	0.038	0.942	0.312	0.043	0.040	0.882
*NNMI*_*CLR*_(5,0.4,0.4;0.2)	0.390	0.035	0.038	0.954	0.307	0.040	0.039	0.912
*NNMI*_*CLR*_(5,0.1,0.7;0.2)	0.377	0.037	0.039	0.940	0.307	0.041	0.040	0.924
*NNMI*_*CLR*_(5,0.7,0.1;0.2)	0.401	0.036	0.037	0.938	0.313	0.041	0.039	0.912

Est: Estimates of probabilities; SD: Empirical standard deviation; SE: Estimate of standard error; CR: Coverage rate of 95% confidence intervals; FO: fully observed; CC: Complete Cases; CE: Calibration estimator; PMI: Parametric Multiple Imputation; *NNMI*_*MLR*_(*NN*,*ω*_1_,*ω*_2_;*ω*_3_): the NNMI method using Multinomial Logistic Regressions, NN is the number of nearest neighbors and weights are *ω*_1_,*ω*_2_, and *ω*_3_; *NNMI*_*CLR*_: the NNMI method using Cumulative Logistic Regressions; *K* = 10 imputed datasets are used for PMI and NNMI methods

Tables 3, 4 and 5 include results when the working models use misspecified link functions, when the missingness probabilities are not extreme. In Table 3, a probit link function is used to generate *Y* and a logit link is used to generate *δ*. When the link function for the working outcome model is misspecified as a logit function, NNMI performs slightly better than CE and PMI (more apparently for estimating *Pr*(*Y*=2) with smaller biases and SDs. In Table 4, a logit link function is used to generate *Y* and a probit link function is used to generate *δ*. When the link function for the working propensity model is misspecified as a probit function, NNMI methods produce slightly larger biases than CE and PMI, yet with smaller SDs and SEs, compared with CE and PMI. In Table 5, probit link functions are used to generate both *Y* and *δ*. When both working models employ logit link functions, CE produces good CRs yet relatively large SDs and SEs. PMI degrades with larger biases and low CRs, more apparently for estimating *Pr*(*Y*=2). NNMI produces more robust results with smaller biases, lower SDs and SEs, and good CRs. For data with more extreme missingness probabilities (See Additional file 1), the comparative pattern remain similar but all methods perform worse.

**Table 3:** Simulation results from probability estimation for *Y*, where *Y* is generated using a probit link function with five covariates, *δ* is generated using a logit link function with not extreme missingness probabilities (M1) based on five covariates, N = 400

	*Pr*(*Y*=1)=0.297				*Pr*(*Y*=2)=0.250			
Method	Est	SD	SE	CR	Est	SD	SE	CR
FO	0.298	0.023	0.023	0.952	0.249	0.021	0.022	0.974
CC	0.322	0.032	0.033	0.910	0.303	0.033	0.032	0.606
	Working models for *Y*:				Five covariates with logit link			
					(misspecified scenario 3)			
	Working models for *δ*:				Five covariates with logit link			
CE	0.291	0.036	0.037	0.954	0.230	0.031	0.032	0.900
PMI	0.307	0.033	0.033	0.942	0.271	0.034	0.033	0.902
*NNMI*_*MLR*_(5,0.4,0.4;0.2)	0.301	0.033	0.033	0.940	0.260	0.031	0.032	0.942
*NNMI*_*MLR*_(5,0.1,0.7;0.2)	0.302	0.034	0.034	0.946	0.259	0.032	0.032	0.936
*NNMI*_*MLR*_(5,0.7,0.1;0.2)	0.301	0.032	0.033	0.944	0.260	0.033	0.032	0.930
*NNMI*_*CLR*_(5,0.4,0.4;0.2)	0.299	0.033	0.033	0.936	0.263	0.032	0.033	0.936
*NNMI*_*CLR*_(5,0.1,0.7;0.2)	0.297	0.033	0.033	0.930	0.263	0.033	0.033	0.926
*NNMI*_*CLR*_(5,0.7,0.1;0.2)	0.302	0.032	0.034	0.948	0.261	0.032	0.032	0.942

Est: Estimates of probabilities; SD: Empirical standard deviation; SE: Estimate of standard error; CR: Coverage rate of 95% confidence intervals; FO: fully observed; CC: Complete Cases; CE: Calibration estimator; PMI: Parametric Multiple Imputation; *NNMI*_*MLR*_(*NN*,*ω*_1_,*ω*_2_;*ω*_3_): the NNMI method using Multinomial Logistic Regressions, NN is the number of nearest neighbors and weights are *ω*_1_,*ω*_2_, and *ω*_3_; *NNMI*_*CLR*_: the NNMI method using Cumulative Logistic Regressions; *K* = 10 imputed datasets are used for PMI and NNMI methods

**Table 4:** Simulation results from probability estimation for *Y*, where *Y* is generated using a logit link function with five covariates, *δ* is generated using a probit link function with not extreme missingness probabilities (M1) based on five covariates, N = 400

	*Pr*(*Y*=1)=0.386				*Pr*(*Y*=2)=0.288			
Method	Est	SD	SE	CR	Est	SD	SE	CR
FO	0.386	0.023	0.024	0.960	0.286	0.023	0.023	0.934
CC	0.456	0.033	0.035	0.512	0.357	0.033	0.034	0.472
	Working models for *Y*:				Five covariates with logit link			
	Working models for *δ*:				Five covariates with logit link			
					(misspecified scenario 4)			
CE	0.386	0.056	0.051	0.944	0.287	0.060	0.051	0.910
PMI	0.388	0.033	0.034	0.950	0.288	0.035	0.034	0.926
*NNMI*_*MLR*_(5,0.4,0.4;0.2)	0.391	0.036	0.038	0.954	0.294	0.040	0.039	0.942
*NNMI*_*MLR*_(5,0.1,0.7;0.2)	0.397	0.038	0.041	0.948	0.291	0.039	0.038	0.928
*NNMI*_*MLR*_(5,0.7,0.1;0.2)	0.388	0.035	0.037	0.966	0.299	0.042	0.041	0.928
*NNMI*_*CLR*_(5,0.4,0.4;0.2)	0.387	0.035	0.036	0.948	0.303	0.040	0.041	0.928
*NNMI*_*CLR*_(5,0.1,0.7;0.2)	0.379	0.035	0.036	0.938	0.304	0.040	0.041	0.930
*NNMI*_*CLR*_(5,0.7,0.1;0.2)	0.395	0.036	0.037	0.956	0.302	0.041	0.040	0.924

Est: Estimates of probabilities; SD: Empirical standard deviation; SE: Estimate of standard error; CR: Coverage rate of 95% confidence intervals; FO: fully observed; CC: Complete Cases; CE: Calibration estimator; PMI: Parametric Multiple Imputation; *NNMI*_*MLR*_(*NN*,*ω*_1_,*ω*_2_;*ω*_3_): the NNMI method using Multinomial Logistic Regressions, NN is the number of nearest neighbors and weights are *ω*_1_,*ω*_2_, and *ω*_3_; *NNMI*_*CLR*_: the NNMI method using Cumulative Logistic Regressions; *K* = 10 imputed datasets are used for PMI and NNMI methods

**Table 5:** Simulation results from probability estimation for *Y*, where *Y* is generated using a probit link function with five covariates, *δ* is generated using a probit link function with not extreme missingness probabilities (M1) based on five covariates, N = 400

	*Pr*(*Y*=1)=0.297				*Pr*(*Y*=2)=0.250			
Method	Est	SD	SE	CR	Est	SD	SE	CR
FO	0.298	0.023	0.023	0.952	0.249	0.021	0.022	0.974
CC	0.328	0.032	0.033	0.862	0.323	0.033	0.033	0.406
	Working models for *Y*:				Five covariates with logit link			
					(misspecified scenario 5)			
	Working models for *δ*:				Five covariates with logit link			
					(misspecified scenario 5)			
CE	0.295	0.068	0.058	0.956	0.218	0.049	0.051	0.926
PMI	0.316	0.038	0.038	0.912	0.294	0.038	0.038	0.800
*NNMI*_*MLR*_(5,0.4,0.4;0.2)	0.310	0.039	0.040	0.940	0.275	0.036	0.039	0.930
*NNMI*_*MLR*_(5,0.1,0.7;0.2)	0.314	0.041	0.041	0.934	0.274	0.037	0.038	0.924
*NNMI*_*MLR*_(5,0.7,0.1;0.2)	0.309	0.040	0.040	0.924	0.276	0.038	0.038	0.914
*NNMI*_*CLR*_(5,0.4,0.4;0.2)	0.308	0.040	0.040	0.936	0.279	0.037	0.038	0.924
*NNMI*_*CLR*_(5,0.1,0.7;0.2)	0.305	0.039	0.040	0.930	0.279	0.037	0.039	0.914
*NNMI*_*CLR*_(5,0.7,0.1;0.2)	0.310	0.040	0.040	0.920	0.276	0.037	0.038	0.924

Est: Estimates of probabilities; SD: Empirical standard deviation; SE: Estimate of standard error; CR: Coverage rate of 95% confidence intervals; FO: fully observed; CC: Complete Cases; CE: Calibration estimator; PMI: Parametric Multiple Imputation; *NNMI*_*MLR*_(*NN*,*ω*_1_,*ω*_2_;*ω*_3_): the NNMI method using Multinomial Logistic Regressions, NN is the number of nearest neighbors and weights are *ω*_1_,*ω*_2_, and *ω*_3_; *NNMI*_*CLR*_: the NNMI method using Cumulative Logistic Regressions; *K* = 10 imputed datasets are used for PMI and NNMI methods

In summary, the NNMI strategy can well accommodate misspecified working models when missingness probabilities are not extreme. PMI can break down if its working outcome model is misspecified. When missingness probabilities for some observations are extreme, CE estimates tend to have considerably higher SDs and SEs, while those from NNMI tend to be more stable. Between the two NNMI strategies, NNMI using multinomial logistic/probit regression models performs better than NNMI using cumulative logistic/probit regressions. This might be due to the fact that the data-generating models for *Y* in the simulation follow the multinomial regression scheme. In addition, there exists no apparent effect of specifying different weights for *ω*_1_ and *ω*_2_, as long as *ω*_3_>0.

### Data example

We illustrate the proposed approach using an analysis of 2013 Behavioral Risk Factor Surveillance System (BRFSS) survey sample data [3]. Established in 1984, BRFSS is a nation-wide system of health-related telephone surveys that annually collects state data about U.S. residents regarding their health-related risk and preventive behaviors, health conditions, and information about health services. In this analysis, we are interested in the satisfaction level with health care received for the Hispanic population who were unable to work and had annual household income less than 15000 dollars. From the public-use BRFSS data system, a subset of 1430 participants are selected with fully-observed data of potentially associated covariates. More specifically, this question, the outcome of interest *Y*, consists of 3 categories: 1, Very satisfied (n=624); 2, Somewhat satisfied (n=357); and 3, Not at all satisfied (n=86). To demonstrated the method, we considered to treat those participants who answered “Don’t know/not sure”, “Not applicable”, “Refused”, and those not asked or missing as missing data (*δ*=0, *n*=363). In reality those who answered “Don’t know/not sure”, “Not applicable”, “Refused” are not necessary to be missing. In this example, the overall missingness rate is 25.4%.

We conduct some exploratory analyses to select predictors for this outcome and the missingness indicator (*δ*) using a multinomial logistic regression model and binary logistic regression model, respectively. From these analyses, the satisfaction levels of health care received is shown to be significantly associated with gender, general health, education level, having health care coverage, and having delayed getting medical care. These five covariates are used to fit a multinomial logistic regression model for predicting the satisfaction levels of health care. On the other hand, the missingness indicator is significantly associated with general health, education level, having health care coverage, and having delayed getting medical care. The four variables are included in a logistic regression model for predicting the missingness probability.

Table 6 shows the results by applying different missing data methods for estimating the marginal distribution of *Y*. For simplicity, we only list the estimates for the category of “Very satisfied” and “Somewhat satisfied”. Compared with CC, the estimates of CE, PMI and NNMI all give lower probabilities for “Very satisfied” and higher probabilities for “Somewhat satisfied”. It is probable that participants who received excellent health care are more likely to respond to this question, and thus CC overestimates the probability for “Very satisfied” and underestimates those for the remaining groups. The estimates from CE, PMI, and NNMI are largely similar, and thus they provide some robustness check against potential model misspecifications.

**Table 6:** 2013 BRFSS Survey Data: Estimation for the probabilities of satisfaction with health care received for the Hispanic participants who were unable to work with annual household income less than 15000 dollars, N=1430 (overall missing rate=25.4%)

	*Pr*(*Y*=Very Satisfied)		*Pr*(*Y*=Somewhat Satisfied)	
Method	Est (SE)	95% CI	Est (SE)	95% CI
CC	0.585 (0.015)	(0.555, 0.614)	0.335 (0.014)	(0.306, 0.363)
CE	0.553 (0.016)	(0.521, 0.584)	0.349 (0.016)	(0.319, 0.380)
PMI	0.552 (0.014)	(0.524, 0.581)	0.345 (0.014)	(0.318, 0.372)
*NNMI*_*MLR*_(5,0.4,0.4;0.2)	0.560 (0.019)	(0.522, 0.598)	0.353 (0.020)	(0.314, 0.392)
*NNMI*_*MLR*_(5,0.1,0.7;0.2)	0.556 (0.019)	(0.519, 0.592)	0.351 (0.021)	(0.310, 0.391)
*NNMI*_*MLR*_(5,0.7,0.1;0.2)	0.550 (0.022)	(0.507, 0.594)	0.359 (0.019)	(0.322, 0.396)
*NNMI*_*CLR*_(5,0.4,0.4;0.2)	0.547 (0.021)	(0.506, 0.588)	0.358 (0.017)	(0.324, 0.392)
*NNMI*_*CLR*_(5,0.1,0.7;0.2)	0.559 (0.016)	(0.528, 0.590)	0.352 (0.016)	(0.320, 0.383)
*NNMI*_*CLR*_(5,0.7,0.1;0.2)	0.555 (0.018)	(0.520, 0.590)	0.350 (0.019)	(0.314, 0.387)

Est: Estimates of probabilities; SE: Estimate of standard error; 95%CI: 95% confidence interval

**X**: covariates as gender, general health, education level, having health care coverage, and having delayed getting medical care, that are used in working models

CC: Complete Cases; CE: Calibration estimator; PMI: Parametric Multiple Imputation; *NNMI*_*MLR*_(*NN*,*ω*_1_,*ω*_2_;*ω*_3_): denotes the NNMI method using Multinomial Logistic Regressions, NN is the number of nearest neighbors and weights are *ω*_1_,*ω*_2_, and *ω*_3_; *NNMI*_*CLR*_: the NNMI method using Cumulative Logistic Regressions; *K* = 10 imputed datasets are used for PMI and NNMI methods

Note that this simple example is merely used to illustrate the proposed statistical methodology, and the results should not be considered for subject-matter interests. More in-depth analyses targeted for the latter purpose should follow the guidelines provided in [3].

## Discussion

In this article, we investigate a nearest-neighbour multiple imputation procedure for missing categorical data. The method applies predictive working models to identify observed cases in the neighbors for each missing observation. The use of information from two working models, one for *Y* and one for *δ*, might result in a double robustness property, which induces consistent estimates even if one of the two working models is misspecified. We use two types of modeling strategies for *Y* with more than two categories, one multinomial logistic/probit regression model and the other is based on *m*−1 cumulative logistic/probit regression models. The results show some but not significant differences between the two strategies, indicating the flexibility of NNMI in terms of the modeling choices. The simulation results also suggest that the proposed approach in general yield satisfactory performances. The setup of the weighted sum of predictive scores would facilitate some sensitivity analyses.

In simulation, we observe that the correctness of the working model for predicting missingness probabilities might be more important than that for the outcome model for categorical *Y*. This implies that a non-zero weight (*ω*_3_) should be applied to the predictive scores from the working model for missingness probabilities. Therefore, it might be more important to seek good models for predicting missingness probabilities for categorical *Y*, compared with continuous *Y*.

The CE results can be unstable with high SEs when missingness rates are relatively high. This is because when the number of complete cases is small, the working model fitted for *Y* might not be accurate and so would negatively affect CE. In contrast, the NNMI estimates tend to be more robust, possibly due to less reliance on the working models for *Y* than CE and instead using the nearest-neighbour approach. In addition, CE performs badly with high SDs when the missingness probabilities were close to 0 or 1, while NNMI suffers less from this problem.

Furthermore, whether the working models, especially the one for the outcome, are correctly specified does not substantially impact NNMI if proper weights are specified. However, CE is more sensitive to the specification of the working model for the categorical variable with missing values. Among all tested specifications, the simulation results suggest that a multinomial logistic/probit regression for predicting missing values and a non-zero weight on the missingness probability predictive score, e.g. *NNMI*_*MLR*_(0.4,0.4;0.2), are preferred to impute categorical data with three or more levels in the absence of prior knowledge on the working models.

This study does not compare results using different *NN* (number of donors). Further research can be conducted for selecting the optimal size of the nearest neighborhood. Another extension is to apply NNMI to impute missing continuous and categorical data simultaneously. Also, the next step can assess the estimation of regression coefficients as well as the performance on imputing missing categorical covariates.

We have demonstrated that the proposed NNMI approach can be applied on missing at random categorical outcome variables with more than two levels for estimating the marginal mean, which broadly exist in public health studies, e.g., health status, health disparity and quality of life. For example, in a study of caries-risk [1], 97 out of 577 subjects dropped out during the recruiting procedure. Among the dropouts, 82 did not attend the examination and 15 did not completely fill out the questionnaire. It was doubtful that the missing mechanism for the 82 subjects was Missing Complete at Random, since subjects with caries were less likely to attend the examination. If the missingness was MAR, then CC would be valid for estimating the association with caries risk in their Table 3 but invalid for estimating the proportions of caries risk levels in their Table 2. Instead of using complete cases, the proposed NNMI approach could be applied to impute the caries risk level for the 82 subjects. Therefore, after the imputed data is obtained, the sample size could increase to 562, and it could help estimate the distribution of the caries risk level (low, medium, or high). Considering that the missing rate is not high (14.6%), 10-time MI could be sufficient to perform the imputation– according to a rule of thumb by Rubin [13]– with a multinomial logistic regression for predicting the missing values and a logistic regression model for predicting the missingness probability with non-zero weights, e.g., (0.4,0.4;0.2). The number of donor could be chosen as 5, based on previous studies [8, 11]. For studies with a higher missing rate, the number of multiple imputations might need to increase to the missing rate in order to achieve both reliable point esimates and reliable standard errors, according to a rule of thumb described by Hippel [18]. Therefore, the proposed approach is applicable for researchers with an interest in assessing the distribution of a categorical outcome with MAR values.

## Conclusions

In conclusion, the proposed multiple imputation method is a reasonable approach to dealing with missing categorical outcome with more than two levels for evaluating the distribution of the outcome. The NNMI approach can work better than PMI when the working model for missing outcome is wrong. When the missing probabilities are extreme, NNMI performs more stably than CE, which results in relatively larger SE.

## Additional file

Additional file 1 Supplementary materials for ‘A Nonparametric Multiple Imputation Approach for Missing Categorical Data’. Tables with the additional simulation results. (PDF 103 kb)

## Abbreviations

BRFSS: Behavioral risk factor surveillance system
CC: Complete-case
CE: Calibration estimator
CR: Coverage rate
EM: Expectation-maximization
EST: estimate
FO: Fully observed
MI: Multiple imputation
NNMI: Nearest neighbor-based multiple imputation
PMI: Parametric multiple imputation
SD: Standard deviation
SE: Standard error
