# Supplementary material for: A nonparametric multiple imputation approach for missing categorical data
Source: BMC Med Res Methodol. 2017 Jun 6;17:87. doi: 10.1186/s12874-017-0360-2 (PMC5461637; doi:10.1186/s12874-017-0360-2)
Supplement: Supplementary file 1 — Supplementary materials for ‘A Nonparametric Multiple Imputation Approach for Missing Categorical Data’. Tables with the additional simulation results. (PDF 103 kb) [file 12874_2017_360_MOESM1_ESM.pdf]

Supplementary Materials for 'A Nonparametric Multiple Imputation Approach for Missing Categorical Data'

Table S1 Simulation results from probability estimation for Y, where Y is generated using a probit link function with five covariates, δ is generated using a logit link function with extreme missingness probabilities (M2) based on five covariates, N = 400

	$Pr(Y=1)$=0.297				$Pr(Y=2)$=0.250			
Method	Est	SD	SE	CR	Est	SD	SE	CR
FO	0.298	0.023	0.023	0.952	0.249	0.021	0.022	0.974
CC	0.310	0.031	0.031	0.938	0.334	0.031	0.032	0.230
			Working models for Y:		Five covariates with logit link (misspecified scenario 3)			
			Working models for δ:		Five covariates with logit link			
CE	0.293	0.151	0.104	0.954	0.218	0.091	0.089	0.968
PMI	0.302	0.039	0.039	0.926	0.306	0.038	0.039	0.696
$NNMI_{MLR}$(5,0.4,0.4;0.2)	0.302	0.042	0.042	0.932	0.281	0.039	0.040	0.904
$NNMI_{MLR}$(5,0.1,0.7;0.2)	0.308	0.044	0.045	0.914	0.279	0.039	0.041	0.922
$NNMI_{MLR}$(5,0.7,0.1;0.2)	0.300	0.042	0.042	0.926	0.284	0.039	0.041	0.908
$NNMI_{CLR}$(5,0.4,0.4;0.2)	0.299	0.042	0.041	0.914	0.285	0.039	0.041	0.910
$NNMI_{CLR}$(5,0.1,0.7;0.2)	0.296	0.043	0.042	0.920	0.285	0.039	0.041	0.894
$NNMI_{CLR}$(5,0.7,0.1;0.2)	0.301	0.042	0.042	0.922	0.284	0.038	0.041	0.914

Est: Estimates of probabilities; SD: Empirical standard deviation; SE: Estimate of standard error; CR: Coverage rate of 95% confidence intervals; FO: fully observed; CC: Complete Cases; CE: Calibration estimator; PMI: Parametric Multiple Imputation; $NNMI_{MLR}(NN,\omega_1,\omega_2;\omega_3)$: the NNMI method using Multinomial Logistic Regressions, NN is the number of nearest neighbors and weights are ω_1,ω_2, and ω_3; $NNMI_{CLR}$: the NNMI method using Cumulative Logistic Regressions; K = 10 imputed datasets are used for PMI and NNMI methods.

Table S2 Simulation results from probability estimation for Y, where Y is generated using a logit link function with five covariates, δ is generated using a probit link function with extreme missingness probabilities (M2) based on five covariates, N = 400

	$Pr(Y=1)$=0.386				$Pr(Y=2)$=0.288			
Method	Est	SD	SE	CR	Est	SD	SE	CR
FO	0.386	0.023	0.024	0.960	0.286	0.023	0.023	0.934
CC	0.429	0.031	0.033	0.782	0.381	0.033	0.033	0.184
			Working models for Y:		Five covariates with logit link			
			Working models for δ:		Five covariates with logit link (misspecified scenario 4)			
CE	0.386	0.122	0.098	0.970	0.282	0.080	0.078	0.922
PMI	0.386	0.035	0.038	0.966	0.289	0.037	0.035	0.922
$NNMI_{MLR}$(5,0.4,0.4;0.2)	0.396	0.044	0.048	0.946	0.304	0.048	0.047	0.906
$NNMI_{MLR}$(5,0.1,0.7;0.2)	0.415	0.051	0.055	0.918	0.298	0.047	0.046	0.898
$NNMI_{MLR}$(5,0.7,0.1;0.2)	0.390	0.042	0.045	0.944	0.313	0.051	0.050	0.914
$NNMI_{CLR}$(5,0.4,0.4;0.2)	0.388	0.040	0.043	0.952	0.313	0.049	0.048	0.892
$NNMI_{CLR}$(5,0.1,0.7;0.2)	0.371	0.042	0.044	0.916	0.318	0.049	0.049	0.912
$NNMI_{CLR}$(5,0.7,0.1;0.2)	0.401	0.041	0.044	0.936	0.315	0.048	0.048	0.918

Est: Estimates of probabilities; SD: Empirical standard deviation; SE: Estimate of standard error; CR: Coverage rate of 95% confidence intervals; FO: fully observed; CC: Complete Cases; CE: Calibration estimator; PMI: Parametric Multiple Imputation; $NNMI_{MLR}(NN, \omega_1, \omega_2; \omega_3)$: the NNMI method using Multinomial Logistic Regressions, NN is the number of nearest neighbors and weights are ω_1, ω_2, and ω_3; $NNMI_{CLR}$: the NNMI method using Cumulative Logistic Regressions; K = 10 imputed datasets are used for PMI and NNMI methods.

Table S3 Simulation results from probability estimation for Y, where Y is generated using a probit link function with five covariates, δ is generated using a probit link function with extreme missingness probabilities (M2) based on five covariates, N = 400

	$Pr(Y=1)$=0.297				$Pr(Y=2)$=0.250			
Method	Est	SD	SE	CR	Est	SD	SE	CR
FO	0.298	0.023	0.023	0.952	0.249	0.021	0.022	0.974
CC	0.310	0.031	0.031	0.924	0.343	0.031	0.032	0.156
			Working models for Y:		Five covariates with logit link (misspecified scenario 5)			
			Working models for δ:		Five covariates with logit link (misspecified scenario 5)			
CE	0.311	0.131	0.106	0.964	0.274	0.123	0.105	0.934
PMI	0.301	0.042	0.042	0.932	0.323	0.043	0.044	0.616
$NNMI_{MLR}$(5,0.4,0.4;0.2)	0.305	0.049	0.050	0.940	0.300	0.047	0.050	0.870
$NNMI_{MLR}$(5,0.1,0.7;0.2)	0.312	0.050	0.053	0.948	0.297	0.047	0.051	0.908
$NNMI_{MLR}$(5,0.7,0.1;0.2)	0.302	0.048	0.050	0.928	0.301	0.047	0.050	0.874
$NNMI_{CLR}$(5,0.4,0.4;0.2)	0.302	0.048	0.050	0.936	0.302	0.047	0.050	0.864
$NNMI_{CLR}$(5,0.1,0.7;0.2)	0.301	0.049	0.051	0.948	0.302	0.048	0.050	0.854
$NNMI_{CLR}$(5,0.7,0.1;0.2)	0.303	0.049	0.050	0.930	0.303	0.047	0.051	0.876

Est: Estimates of probabilities; SD: Empirical standard deviation; SE: Estimate of standard error; CR: Coverage rate of 95% confidence intervals; FO: fully observed; CC: Complete Cases; CE: Calibration estimator; PMI: Parametric Multiple Imputation; $NNMI_{MLR}(NN, \omega_1, \omega_2; \omega_3)$: the NNMI method using Multinomial Logistic Regressions, NN is the number of nearest neighbors and weights are ω_1, ω_2, and ω_3; $NNMI_{CLR}$: the NNMI method using Cumulative Logistic Regressions; K = 10 imputed datasets are used for PMI and NNMI methods.

Table S4 Simulation results from probability estimation for Y, where Y is generated using a logit link function with five covariates, δ is generated using a logit link function with not extreme missingness probabilities (M1) based on five covariates, N = 200

	$Pr(Y=1)=0.386$				$Pr(Y=2)=0.288$			
Method	Est	SD	SE	CR	Est	SD	SE	CR
FO	0.388	0.035	0.034	0.950	0.286	0.033	0.032	0.936
CC	0.443	0.052	0.050	0.792	0.338	0.051	0.047	0.804
				Working models for Y:	Five covariates with logit link			
				Working models for δ:	Five covariates with logit link			
CE	0.387	0.052	0.053	0.952	0.286	0.056	0.054	0.926
PMI	0.388	0.046	0.045	0.942	0.287	0.048	0.045	0.924
$NNMI_{MLR}$(5,0.4,0.4;0.2)	0.390	0.048	0.047	0.936	0.291	0.050	0.048	0.932
$NNMI_{MLR}$(5,0.1,0.7;0.2)	0.396	0.049	0.050	0.940	0.288	0.049	0.047	0.932
$NNMI_{MLR}$(5,0.7,0.1;0.2)	0.388	0.048	0.047	0.940	0.294	0.052	0.048	0.922
$NNMI_{CLR}$(5,0.4,0.4;0.2)	0.386	0.047	0.047	0.964	0.300	0.052	0.049	0.926
$NNMI_{CLR}$(5,0.1,0.7;0.2)	0.381	0.048	0.047	0.920	0.300	0.052	0.049	0.908
$NNMI_{CLR}$(5,0.7,0.1;0.2)	0.393	0.047	0.047	0.942	0.300	0.052	0.049	0.930
				Working models for Y:	Three covariates with logit link (misspecified scenario 1)			
				Working models for δ:	Five covariates with logit link			
CE	0.306	0.084	0.095	0.944	0.288	0.061	0.062	0.942
PMI	0.466	0.055	0.054	0.672	0.285	0.047	0.044	0.912
$NNMI_{MLR}$(5,0.4,0.4;0.2)	0.419	0.049	0.055	0.924	0.292	0.050	0.047	0.926
$NNMI_{MLR}$(5,0.1,0.7;0.2)	0.418	0.050	0.055	0.924	0.290	0.049	0.047	0.922
$NNMI_{MLR}$(5,0.7,0.1;0.2)	0.418	0.050	0.055	0.920	0.294	0.050	0.048	0.940
$NNMI_{CLR}$(5,0.4,0.4;0.2)	0.424	0.050	0.054	0.898	0.295	0.050	0.047	0.918
$NNMI_{CLR}$(5,0.1,0.7;0.2)	0.419	0.050	0.054	0.912	0.295	0.050	0.048	0.932
$NNMI_{CLR}$(5,0.7,0.1;0.2)	0.424	0.050	0.055	0.916	0.295	0.050	0.048	0.932
				Working models for Y:	Five covariates with logit link			
				Working models for δ:	Three covariates with logit link (misspecified scenario 2)			
CE	0.389	0.048	0.048	0.944	0.285	0.051	0.047	0.934
PMI	0.388	0.046	0.045	0.942	0.287	0.048	0.045	0.924
$NNMI_{MLR}$(5,0.4,0.4;0.2)	0.396	0.048	0.048	0.932	0.294	0.051	0.048	0.930
$NNMI_{MLR}$(5,0.1,0.7;0.2)	0.411	0.050	0.051	0.936	0.290	0.049	0.046	0.908
$NNMI_{MLR}$(5,0.7,0.1;0.2)	0.392	0.048	0.047	0.932	0.301	0.052	0.049	0.924
$NNMI_{CLR}$(5,0.4,0.4;0.2)	0.390	0.047	0.047	0.930	0.301	0.051	0.047	0.926
$NNMI_{CLR}$(5,0.1,0.7;0.2)	0.384	0.049	0.047	0.936	0.298	0.050	0.047	0.928
$NNMI_{CLR}$(5,0.7,0.1;0.2)	0.396	0.047	0.047	0.944	0.307	0.051	0.048	0.920

Est: Estimates of probabilities; SD: Empirical standard deviation; SE: Estimate of standard error; CR: Coverage rate of 95% confidence intervals; FO: fully observed; CC: Complete Cases; CE: Calibration estimator; PMI: Parametric Multiple Imputation; $NNMI_{MLR}(NN, \omega_1, \omega_2; \omega_3)$: the NNMI method using Multinomial Logistic Regressions, NN is the number of nearest neighbors and weights are ω_1, ω_2, and ω_3; $NNMI_{CLR}$: the NNMI method using Cumulative Logistic Regressions; K = 10 imputed datasets are used for PMI and NNMI methods.

Table S5 Simulation results from probability estimation for Y, where Y is generated using a probit link function with five covariates, δ is generated using a logit link function with not extreme missingness probabilities (M1) based on five covariates, N = 200

	$Pr(Y=1)$=0.297				$Pr(Y=2)$=0.250			
Method	Est	SD	SE	CR	Est	SD	SE	CR
FO	0.297	0.033	0.032	0.950	0.248	0.030	0.030	0.956
CC	0.317	0.044	0.046	0.944	0.303	0.046	0.046	0.812
				Working models for Y:	Five covariates with logit link (misspecified scenario 3)			
				Working models for δ:	Five covariates with logit link			
CE	0.289	0.052	0.058	0.964	0.228	0.046	0.052	0.938
PMI	0.306	0.047	0.049	0.960	0.270	0.046	0.047	0.946
$NNMI_{MLR}$(5,0.4,0.4;0.2)	0.300	0.048	0.049	0.960	0.264	0.048	0.046	0.940
$NNMI_{MLR}$(5,0.1,0.7;0.2)	0.302	0.048	0.050	0.950	0.263	0.048	0.046	0.960
$NNMI_{MLR}$(5,0.7,0.1;0.2)	0.300	0.047	0.049	0.944	0.266	0.046	0.047	0.948
$NNMI_{CLR}$(5,0.4,0.4;0.2)	0.300	0.047	0.050	0.954	0.268	0.047	0.047	0.946
$NNMI_{CLR}$(5,0.1,0.7;0.2)	0.297	0.048	0.050	0.944	0.269	0.047	0.048	0.938
$NNMI_{CLR}$(5,0.7,0.1;0.2)	0.301	0.049	0.049	0.936	0.269	0.048	0.047	0.948

Est: Estimates of probabilities; SD: Empirical standard deviation; SE: Estimate of standard error; CR: Coverage rate of 95% confidence intervals; FO: fully observed; CC: Complete Cases; CE: Calibration estimator; PMI: Parametric Multiple Imputation; $NNMI_{MLR}(NN, \omega_1, \omega_2; \omega_3)$: the NNMI method using Multinomial Logistic Regressions, NN is the number of nearest neighbors and weights are ω_1, ω_2, and ω_3; $NNMI_{CLR}$: the NNMI method using Cumulative Logistic Regressions; K = 10 imputed datasets are used for PMI and NNMI methods.

Table S6 Simulation results from probability estimation for Y, where Y is generated using a logit link function with five covariates, δ is generated using a probit link function with not extreme missingness probabilities (M1) based on five covariates, N = 200

	$Pr(Y=1)$=0.386				$Pr(Y=2)$=0.288			
Method	Est	SD	SE	CR	Est	SD	SE	CR
FO	0.388	0.035	0.034	0.950	0.286	0.033	0.032	0.936
CC	0.458	0.051	0.050	0.678	0.357	0.051	0.048	0.696
Working models for Y:					Five covariates with logit link			
Working models for δ:					Five covariates with logit link (misspecified scenario 4)			
CE	0.386	0.073	0.077	0.974	0.287	0.082	0.084	0.936
PMI	0.388	0.049	0.049	0.960	0.289	0.050	0.049	0.940
$NNMI_{MLR}$(5,0.4,0.4;0.2)	0.393	0.052	0.053	0.948	0.299	0.056	0.054	0.926
$NNMI_{MLR}$(5,0.1,0.7;0.2)	0.405	0.054	0.058	0.942	0.293	0.053	0.053	0.922
$NNMI_{MLR}$(5,0.7,0.1;0.2)	0.389	0.051	0.052	0.942	0.304	0.056	0.056	0.934
$NNMI_{CLR}$(5,0.4,0.4;0.2)	0.389	0.050	0.051	0.952	0.309	0.056	0.056	0.946
$NNMI_{CLR}$(5,0.1,0.7;0.2)	0.380	0.051	0.052	0.924	0.308	0.056	0.055	0.926
$NNMI_{CLR}$(5,0.7,0.1;0.2)	0.399	0.051	0.052	0.948	0.310	0.055	0.056	0.930

Est: Estimates of probabilities; SD: Empirical standard deviation; SE: Estimate of standard error; CR: Coverage rate of 95% confidence intervals; FO: fully observed; CC: Complete Cases; CE: Calibration estimator; PMI: Parametric Multiple Imputation; $NNMI_{MLR}(NN, \omega_1, \omega_2; \omega_3)$: the NNMI method using Multinomial Logistic Regressions, NN is the number of nearest neighbors and weights are ω_1, ω_2, and ω_3; $NNMI_{CLR}$: the NNMI method using Cumulative Logistic Regressions; K = 10 imputed datasets are used for PMI and NNMI methods.

Table S7 Simulation results from probability estimation for Y, where Y is generated using a probit link function with five covariates, δ is generated using a probit link function with not extreme missingness probabilities (M1) based on five covariates, N = 200

	$Pr(Y=1)$=0.297				$Pr(Y=2)$=0.250			
Method	Est	SD	SE	CR	Est	SD	SE	CR
FO	0.297	0.033	0.032	0.950	0.248	0.030	0.030	0.956
CC	0.323	0.044	0.047	0.928	0.324	0.047	0.047	0.692
Working models for Y:					Five covariates with logit link (misspecified scenario 5)			
Working models for δ:					Five covariates with logit link (misspecified scenario 5)			
CE	0.298	0.098	0.105	0.968	0.217	0.078	0.097	0.966
PMI	0.315	0.054	0.056	0.930	0.297	0.054	0.055	0.894
$NNMI_{MLR}$(5,0.4,0.4;0.2)	0.311	0.056	0.057	0.928	0.285	0.054	0.056	0.932
$NNMI_{MLR}$(5,0.1,0.7;0.2)	0.315	0.056	0.059	0.952	0.283	0.054	0.055	0.928
$NNMI_{MLR}$(5,0.7,0.1;0.2)	0.311	0.056	0.058	0.934	0.285	0.052	0.056	0.950
$NNMI_{CLR}$(5,0.4,0.4;0.2)	0.311	0.056	0.058	0.946	0.289	0.053	0.055	0.910
$NNMI_{CLR}$(5,0.1,0.7;0.2)	0.308	0.055	0.057	0.934	0.288	0.051	0.055	0.926
$NNMI_{CLR}$(5,0.7,0.1;0.2)	0.313	0.058	0.059	0.932	0.288	0.053	0.056	0.932

Est: Estimates of probabilities; SD: Empirical standard deviation; SE: Estimate of standard error; CR: Coverage rate of 95% confidence intervals; FO: fully observed; CC: Complete Cases; CE: Calibration estimator; PMI: Parametric Multiple Imputation; $NNMI_{MLR}(NN, \omega_1, \omega_2; \omega_3)$: the NNMI method using Multinomial Logistic Regressions, NN is the number of nearest neighbors and weights are ω_1, ω_2, and ω_3; $NNMI_{CLR}$: the NNMI method using Cumulative Logistic Regressions; K = 10 imputed datasets are used for PMI and NNMI methods.

Table S8 Simulation results from probability estimation for Y, where Y is generated using a logit link function with five covariates, δ is generated using a logit link function with extreme missingness probabilities (M2) based on five covariates, N = 200

	$Pr(Y=1)$=0.386				$Pr(Y=2)$=0.288			
Method	Est	SD	SE	CR	Est	SD	SE	CR
FO	0.388	0.035	0.034	0.950	0.286	0.033	0.032	0.936
CC	0.428	0.049	0.047	0.846	0.374	0.048	0.046	0.562
			Working models for Y:		Five covariates with logit link			
			Working models for δ:		Five covariates with logit link			
CE	0.382	0.110	0.132	0.964	0.282	0.103	0.120	0.954
PMI	0.387	0.052	0.050	0.918	0.292	0.048	0.047	0.934
$NNMI_{MLR}$(5,0.4,0.4;0.2)	0.390	0.056	0.055	0.920	0.303	0.053	0.054	0.952
$NNMI_{MLR}$(5,0.1,0.7;0.2)	0.405	0.058	0.063	0.948	0.298	0.051	0.053	0.948
$NNMI_{MLR}$(5,0.7,0.1;0.2)	0.387	0.054	0.054	0.934	0.311	0.056	0.057	0.932
$NNMI_{CLR}$(5,0.4,0.4;0.2)	0.387	0.053	0.053	0.936	0.312	0.053	0.055	0.942
$NNMI_{CLR}$(5,0.1,0.7;0.2)	0.374	0.054	0.054	0.906	0.313	0.053	0.056	0.936
$NNMI_{CLR}$(5,0.7,0.1;0.2)	0.398	0.054	0.054	0.942	0.315	0.054	0.057	0.924
			Working models for Y:		Three covariates with logit link (misspecified scenario 1)			
			Working models for δ:		Five covariates with logit link			
CE	0.313	0.273	0.327	0.968	0.283	0.114	0.133	0.964
PMI	0.492	0.059	0.058	0.538	0.291	0.045	0.045	0.936
$NNMI_{MLR}$(5,0.4,0.4;0.2)	0.439	0.061	0.068	0.898	0.301	0.052	0.054	0.944
$NNMI_{MLR}$(5,0.1,0.7;0.2)	0.435	0.061	0.069	0.906	0.299	0.052	0.054	0.938
$NNMI_{MLR}$(5,0.7,0.1;0.2)	0.437	0.061	0.068	0.898	0.305	0.053	0.056	0.940
$NNMI_{CLR}$(5,0.4,0.4;0.2)	0.444	0.058	0.065	0.860	0.304	0.052	0.054	0.940
$NNMI_{CLR}$(5,0.1,0.7;0.2)	0.431	0.057	0.064	0.914	0.308	0.054	0.055	0.944
$NNMI_{CLR}$(5,0.7,0.1;0.2)	0.446	0.061	0.067	0.842	0.304	0.054	0.054	0.930
			Working models for Y:		Five covariates with logit link			
			Working models for δ:		Three covariates with logit link (misspecified scenario 2)			
CE	0.391	0.074	0.075	0.960	0.288	0.065	0.062	0.930
PMI	0.387	0.052	0.050	0.918	0.292	0.048	0.047	0.934
$NNMI_{MLR}$(5,0.4,0.4;0.2)	0.401	0.055	0.055	0.920	0.306	0.050	0.053	0.928
$NNMI_{MLR}$(5,0.1,0.7;0.2)	0.436	0.058	0.061	0.868	0.297	0.049	0.051	0.942
$NNMI_{MLR}$(5,0.7,0.1;0.2)	0.393	0.054	0.053	0.918	0.319	0.054	0.055	0.924
$NNMI_{CLR}$(5,0.4,0.4;0.2)	0.393	0.053	0.052	0.924	0.314	0.051	0.053	0.934
$NNMI_{CLR}$(5,0.1,0.7;0.2)	0.382	0.055	0.053	0.912	0.311	0.050	0.053	0.940
$NNMI_{CLR}$(5,0.7,0.1;0.2)	0.406	0.054	0.053	0.924	0.322	0.052	0.054	0.916

Est: Estimates of probabilities; SD: Empirical standard deviation; SE: Estimate of standard error; CR: Coverage rate of 95% confidence intervals; FO: fully observed; CC: Complete Cases; CE: Calibration estimator; PMI: Parametric Multiple Imputation; $NNMI_{MLR}(NN, \omega_1, \omega_2; \omega_3)$: the NNMI method using Multinomial Logistic Regressions, NN is the number of nearest neighbors and weights are ω_1, ω_2, and ω_3; $NNMI_{CLR}$: the NNMI method using Cumulative Logistic Regressions; K = 10 imputed datasets are used for PMI and NNMI methods.

Table S9 Simulation results from probability estimation for Y, where Y is generated using a probit link function with five covariates, δ is generated using a logit link function with extreme missingness probabilities (M2) based on five covariates, N = 200

	$Pr(Y=1)$=0.297				$Pr(Y=2)$=0.250			
Method	Est	SD	SE	CR	Est	SD	SE	CR
FO	0.297	0.033	0.032	0.950	0.248	0.030	0.030	0.956
CC	0.308	0.043	0.044	0.952	0.333	0.045	0.045	0.560
		Working models for Y:			Five covariates with logit link (misspecified scenario 3)			
		Working models for δ:			Five covariates with logit link			
CE	0.298	0.152	0.210	0.964	0.211	0.138	0.236	0.980
PMI	0.302	0.054	0.055	0.924	0.305	0.053	0.054	0.860
$NNMI_{MLR}$(5,0.4,0.4;0.2)	0.302	0.059	0.059	0.918	0.290	0.054	0.056	0.932
$NNMI_{MLR}$(5,0.1,0.7;0.2)	0.307	0.058	0.061	0.924	0.287	0.056	0.057	0.924
$NNMI_{MLR}$(5,0.7,0.1;0.2)	0.301	0.058	0.059	0.932	0.291	0.054	0.058	0.934
$NNMI_{CLR}$(5,0.4,0.4;0.2)	0.301	0.058	0.059	0.914	0.292	0.053	0.056	0.916
$NNMI_{CLR}$(5,0.1,0.7;0.2)	0.298	0.057	0.058	0.916	0.292	0.054	0.057	0.918
$NNMI_{CLR}$(5,0.7,0.1;0.2)	0.303	0.058	0.060	0.912	0.293	0.054	0.057	0.928

Est: Estimates of probabilities; SD: Empirical standard deviation; SE: Estimate of standard error; CR: Coverage rate of 95% confidence intervals; FO: fully observed; CC: Complete Cases; CE: Calibration estimator; PMI: Parametric Multiple Imputation; $NNMI_{MLR}(NN, \omega_1, \omega_2; \omega_3)$: the NNMI method using Multinomial Logistic Regressions, NN is the number of nearest neighbors and weights are ω_1, ω_2, and ω_3; $NNMI_{CLR}$: the NNMI method using Cumulative Logistic Regressions; K = 10 imputed datasets are used for PMI and NNMI methods.

Table S10 Simulation results from probability estimation for Y, where Y is generated using a logit link function with five covariates, δ is generated using a probit link function with extreme missingness probabilities (M2) based on five covariates, N = 200

	$Pr(Y=1)$=0.386				$Pr(Y=2)$=0.288			
Method	Est	SD	SE	CR	Est	SD	SE	CR
FO	0.388	0.035	0.034	0.950	0.286	0.033	0.032	0.936
CC	0.431	0.049	0.047	0.826	0.380	0.050	0.046	0.498
				Working models for Y:	Five covariates with logit link			
				Working models for δ:	Five covariates with logit link (misspecified scenario 4)			
CE	0.398	0.144	0.154	0.952	0.275	0.156	0.167	0.966
PMI	0.388	0.055	0.054	0.928	0.291	0.049	0.050	0.924
$NNMI_{MLR}$(5,0.4,0.4;0.2)	0.396	0.060	0.062	0.926	0.306	0.056	0.060	0.940
$NNMI_{MLR}$(5,0.1,0.7;0.2)	0.420	0.066	0.071	0.916	0.299	0.055	0.060	0.940
$NNMI_{MLR}$(5,0.7,0.1;0.2)	0.392	0.060	0.060	0.924	0.317	0.059	0.065	0.934
$NNMI_{CLR}$(5,0.4,0.4;0.2)	0.392	0.057	0.058	0.930	0.316	0.055	0.061	0.944
$NNMI_{CLR}$(5,0.1,0.7;0.2)	0.375	0.060	0.060	0.914	0.316	0.057	0.061	0.960
$NNMI_{CLR}$(5,0.7,0.1;0.2)	0.406	0.060	0.059	0.920	0.318	0.059	0.061	0.914

Est: Estimates of probabilities; SD: Empirical standard deviation; SE: Estimate of standard error; CR: Coverage rate of 95% confidence intervals; FO: fully observed; CC: Complete Cases; CE: Calibration estimator; PMI: Parametric Multiple Imputation; $NNMI_{MLR}(NN, \omega_1, \omega_2; \omega_3)$: the NNMI method using Multinomial Logistic Regressions, NN is the number of nearest neighbors and weights are ω_1, ω_2, and ω_3; $NNMI_{CLR}$: the NNMI method using Cumulative Logistic Regressions; K = 10 imputed datasets are used for PMI and NNMI methods.

Table S11 Simulation results from probability estimation for Y, where Y is generated using a probit link function with five covariates, δ is generated using a probit link function with extreme missingness probabilities (M2) based on five covariates, N = 200

	$Pr(Y=1)$=0.297				$Pr(Y=2)$=0.250			
Method	Est	SD	SE	CR	Est	SD	SE	CR
FO	0.297	0.033	0.032	0.950	0.248	0.030	0.030	0.956
CC	0.308	0.044	0.044	0.946	0.341	0.046	0.045	0.474
				Working models for Y:	Five covariates with logit link (misspecified scenario 5)			
				Working models for δ:	Five covariates with logit link (misspecified scenario 5)			
CE	0.318	0.204	0.218	0.956	0.264	0.188	0.245	0.974
PMI	0.303	0.060	0.059	0.920	0.322	0.063	0.061	0.822
$NNMI_{MLR}$(5,0.4,0.4;0.2)	0.306	0.065	0.065	0.930	0.307	0.064	0.065	0.902
$NNMI_{MLR}$(5,0.1,0.7;0.2)	0.312	0.067	0.069	0.936	0.304	0.064	0.066	0.906
$NNMI_{MLR}$(5,0.7,0.1;0.2)	0.303	0.064	0.066	0.928	0.307	0.064	0.065	0.904
$NNMI_{CLR}$(5,0.4,0.4;0.2)	0.304	0.064	0.065	0.916	0.308	0.061	0.063	0.888
$NNMI_{CLR}$(5,0.1,0.7;0.2)	0.305	0.066	0.067	0.924	0.306	0.062	0.065	0.908
$NNMI_{CLR}$(5,0.7,0.1;0.2)	0.305	0.065	0.066	0.914	0.311	0.063	0.066	0.908

Est: Estimates of probabilities; SD: Empirical standard deviation; SE: Estimate of standard error; CR: Coverage rate of 95% confidence intervals; FO: fully observed; CC: Complete Cases; CE: Calibration estimator; PMI: Parametric Multiple Imputation; $NNMI_{MLR}(NN, \omega_1, \omega_2; \omega_3)$: the NNMI method using Multinomial Logistic Regressions, NN is the number of nearest neighbors and weights are ω_1, ω_2, and ω_3; $NNMI_{CLR}$: the NNMI method using Cumulative Logistic Regressions; K = 10 imputed datasets are used for PMI and NNMI methods.

Table S12 Simulation results from probability estimation for Y, where Y is generated using a logit link function with five covariates, δ is generated using a logit link function with not extreme missingness probabilities (M1) based on five covariates, N = 400

	$Pr(Y=1)=0.386$				$Pr(Y=2)=0.288$			
Method	Est	SD	SE	CR	Est	SD	SE	CR
FO	0.386	0.023	0.024	0.960	0.286	0.023	0.023	0.934
CC	0.439	0.034	0.035	0.674	0.340	0.034	0.033	0.670
				Working models for Y:	Five covariates with logit link			
				Working models for δ:	Five covariates with logit link			
CE	0.388	0.036	0.036	0.948	0.286	0.038	0.036	0.924
PMI	0.387	0.030	0.032	0.956	0.287	0.033	0.031	0.946
$NNMI_{MLR}$(5,0.4,0.4;0.2)	0.388	0.031	0.033	0.950	0.288	0.034	0.033	0.942
$NNMI_{MLR}$(5,0.1,0.7;0.2)	0.390	0.033	0.034	0.958	0.287	0.034	0.033	0.932
$NNMI_{MLR}$(5,0.7,0.1;0.2)	0.386	0.031	0.032	0.960	0.291	0.035	0.033	0.942
$NNMI_{CLR}$(5,0.4,0.4;0.2)	0.386	0.031	0.032	0.952	0.295	0.035	0.034	0.936
$NNMI_{CLR}$(5,0.1,0.7;0.2)	0.380	0.032	0.032	0.944	0.295	0.036	0.034	0.938
$NNMI_{CLR}$(5,0.7,0.1;0.2)	0.390	0.031	0.033	0.952	0.294	0.035	0.034	0.938
				Working models for Y:	Three covariates with logit link (misspecified scenario 1)			
				Working models for δ:	Five covariates with logit link			
CE	0.311	0.057	0.057	0.760	0.288	0.041	0.041	0.932
PMI	0.464	0.037	0.038	0.436	0.285	0.032	0.030	0.928
$NNMI_{MLR}$(5,0.4,0.4;0.2)	0.409	0.035	0.038	0.932	0.290	0.034	0.033	0.948
$NNMI_{MLR}$(5,0.1,0.7;0.2)	0.407	0.035	0.038	0.944	0.289	0.034	0.033	0.934
$NNMI_{MLR}$(5,0.7,0.1;0.2)	0.407	0.035	0.038	0.942	0.291	0.034	0.033	0.936
$NNMI_{CLR}$(5,0.4,0.4;0.2)	0.415	0.035	0.038	0.910	0.292	0.034	0.033	0.946
$NNMI_{CLR}$(5,0.1,0.7;0.2)	0.411	0.035	0.038	0.928	0.292	0.034	0.033	0.942
$NNMI_{CLR}$(5,0.7,0.1;0.2)	0.413	0.035	0.038	0.918	0.292	0.034	0.033	0.940
				Working models for Y:	Five covariates with logit link			
				Working models for δ:	Three covariates with logit link (misspecified scenario 2)			
CE	0.389	0.032	0.033	0.954	0.285	0.033	0.032	0.942
PMI	0.387	0.030	0.032	0.956	0.287	0.033	0.031	0.946
$NNMI_{MLR}$(5,0.4,0.4;0.2)	0.392	0.031	0.033	0.962	0.292	0.035	0.033	0.928
$NNMI_{MLR}$(5,0.1,0.7;0.2)	0.402	0.033	0.034	0.954	0.289	0.034	0.032	0.942
$NNMI_{MLR}$(5,0.7,0.1;0.2)	0.390	0.031	0.032	0.958	0.297	0.035	0.033	0.938
$NNMI_{CLR}$(5,0.4,0.4;0.2)	0.387	0.031	0.032	0.950	0.298	0.035	0.033	0.946
$NNMI_{CLR}$(5,0.1,0.7;0.2)	0.382	0.031	0.032	0.946	0.298	0.035	0.033	0.938
$NNMI_{CLR}$(5,0.7,0.1;0.2)	0.392	0.031	0.032	0.958	0.302	0.034	0.033	0.936

Est: Estimates of probabilities; SD: Empirical standard deviation; SE: Estimate of standard error; CR: Coverage rate of 95% confidence intervals; FO: fully observed; CC: Complete Cases; CE: Calibration estimator; PMI: Parametric Multiple Imputation; $NNMI_{MLR}(NN, \omega_1, \omega_2; \omega_3)$: the NNMI method using Multinomial Logistic Regressions, NN is the number of nearest neighbors and weights are ω_1, ω_2, and ω_3; $NNMI_{CLR}$: the NNMI method using Cumulative Logistic Regressions; K = 50 imputed datasets are used for PMI and NNMI methods.

Table S13 Simulation results from NNMI, compared with other methods including Latent Class Model (LCM), from probability estimation for Y, where Y is generated using a logit link function with five covariates, δ is generated using a logit link function with not extreme missingness probabilities (M1) based on five covariates, N = 400

	$Pr(Y=1)=0.386$				$Pr(Y=2)=0.288$			
Method	Est	SD	SE	CR	Est	SD	SE	CR
FO	0.386	0.023	0.024	0.960	0.286	0.023	0.023	0.934
CC	0.439	0.034	0.035	0.674	0.340	0.034	0.033	0.670
				Working models for Y:	Five covariates with logit link			
				Working models for δ:	Five covariates with logit link			
CE	0.388	0.036	0.036	0.948	0.286	0.038	0.036	0.924
PMI	0.387	0.030	0.032	0.954	0.287	0.034	0.032	0.930
LCM	0.396	0.033	0.043	0.978	0.319	0.035	0.046	0.954
$NNMI_{MLR}$(5,0.4,0.4;0.2)	0.387	0.032	0.033	0.952	0.288	0.036	0.033	0.936
$NNMI_{MLR}$(5,0.1,0.7;0.2)	0.389	0.033	0.034	0.956	0.288	0.035	0.033	0.930
$NNMI_{MLR}$(5,0.7,0.1;0.2)	0.386	0.032	0.033	0.956	0.290	0.036	0.034	0.926
$NNMI_{CLR}$(5,0.4,0.4;0.2)	0.385	0.032	0.033	0.948	0.294	0.036	0.034	0.916
$NNMI_{CLR}$(5,0.1,0.7;0.2)	0.381	0.032	0.033	0.944	0.295	0.037	0.034	0.928
$NNMI_{CLR}$(5,0.7,0.1;0.2)	0.390	0.032	0.033	0.950	0.294	0.037	0.034	0.936
				Working models for Y:	Three covariates with logit link (misspecified scenario 1)			
				Working models for δ:	Five covariates with logit link			
CE	0.311	0.057	0.057	0.760	0.288	0.041	0.041	0.932
PMI	0.464	0.037	0.038	0.454	0.285	0.032	0.031	0.922
LCM	0.460	0.038	0.049	0.694	0.299	0.034	0.043	0.974
$NNMI_{MLR}$(5,0.4,0.4;0.2)	0.410	0.036	0.039	0.932	0.290	0.035	0.033	0.926
$NNMI_{MLR}$(5,0.1,0.7;0.2)	0.407	0.036	0.039	0.940	0.290	0.035	0.033	0.932
$NNMI_{MLR}$(5,0.7,0.1;0.2)	0.408	0.035	0.039	0.930	0.291	0.035	0.033	0.928
$NNMI_{CLR}$(5,0.4,0.4;0.2)	0.415	0.036	0.038	0.896	0.292	0.034	0.033	0.940
$NNMI_{CLR}$(5,0.1,0.7;0.2)	0.412	0.036	0.039	0.916	0.292	0.035	0.033	0.934
$NNMI_{CLR}$(5,0.7,0.1;0.2)	0.413	0.035	0.039	0.926	0.291	0.035	0.034	0.954
				Working models for Y:	Five covariates with logit link			
				Working models for δ:	Three covariates with logit link (misspecified scenario 2)			
CE	0.389	0.032	0.033	0.954	0.285	0.033	0.032	0.942
PMI	0.387	0.030	0.032	0.954	0.287	0.034	0.032	0.930
LCM	0.396	0.033	0.043	0.978	0.319	0.035	0.046	0.954
$NNMI_{MLR}$(5,0.4,0.4;0.2)	0.393	0.032	0.033	0.962	0.292	0.035	0.033	0.936
$NNMI_{MLR}$(5,0.1,0.7;0.2)	0.402	0.034	0.035	0.936	0.289	0.035	0.033	0.926
$NNMI_{MLR}$(5,0.7,0.1;0.2)	0.389	0.031	0.033	0.960	0.297	0.036	0.034	0.936
$NNMI_{CLR}$(5,0.4,0.4;0.2)	0.387	0.031	0.032	0.958	0.298	0.035	0.033	0.936
$NNMI_{CLR}$(5,0.1,0.7;0.2)	0.382	0.031	0.033	0.956	0.298	0.035	0.034	0.940
$NNMI_{CLR}$(5,0.7,0.1;0.2)	0.392	0.031	0.033	0.954	0.302	0.035	0.034	0.920

Est: Estimates of probabilities; SD: Empirical standard deviation; SE: Estimate of standard error; CR: Coverage rate of 95% confidence intervals; FO: fully observed; CC: Complete Cases; CE: Calibration estimator; PMI: Parametric Multiple Imputation; LCM: Latent Class Model; $NNMI_{MLR}(NN, \omega_1, \omega_2; \omega_3)$: the NNMI method using Multinomial Logistic Regressions, NN is the number of nearest neighbors and weights are ω_1, ω_2, and ω_3; $NNMI_{CLR}$: the NNMI method using Cumulative Logistic Regressions; K = 10 imputed datasets are used for PMI and NNMI methods.
